# Miscellania

**Published:** 1855-05

**Authors:** 


### MISCELLANIA.

Miss ELIZABETH PRATT, of Boston, has bequeathed $20,000 to the Massachusetts General Hospital.—Dr. LA ROCHE, of Philadelphia, has been appointed to the chair of Pathological Anatomy and Physiology in Memphis Medical College, to fill the vacancy occasioned by the resignation of Dr. O. T. QUINTARD, who designs taking orders in the Episcopal Church. We understand that Dr. LA ROCHE will not accept.—Dr. E. R. PEASLEE has again successfully performed the operation of ovariotomy. The tumor weighed twenty pounds.—The medical class of the University of Buffalo have passed resolutions complimentary to Professors HUNT and WHITE.—The students of the New York Medical College presented Professor BAKER with a very handsome speculum chair.—A Dr. GEORGE BLANCH was recently convicted, in Cincinnati, of manslaughter, for causing the death of a patient, by puncturing a popliteal aneurism.—Professor HARDY V. WOOTEN has retired from the Faculty of the Memphis Medical College.—HENRY ROSSIGNOL, M. D., has become associated with Professor L. A. DUGAS in the editorial management of the Southern Medical and Surgical Journal.—Dr. Bennett Dowler will not issue the Quarterly Journal, the circular of which we have already noticed, the publisher of the New Orleans Medical and Surgical Journal having made an arrangement with him to edit that periodical for a term of five years.—M. Geoffroy St. Hilaire, Professor at the Museum of Natural History, recommends bringing horse flesh into use as food. There is now a privileged slaughter-house for horses at Altort, near Paris, under the control of the Veterinary School where horse flesh is sold at twelve cents per pound. We were once invited to eat some horse steak, but there being beef steak on the table we chose the latter.—Dr. Austin Flint has retired from the Buffalo Medical Journal. It is now conducted by Prof. Sanford B. Hunt, M. D.—Diseases of Women and Children, by Gunning S. Bedford, M. D.; Atlas of Cutaneous Eruptions, by J. Moore Neligan, M. D., M. R. I. A.; The Practitioner's Pharmacopœa and Universal Formulary, by Jno. Foote, M. R. C. S., will soon be issued by S. S. & W. Wood of New York.

---

